# Comparing the Efficacy of Ondansetron, Domperidone, and Metoclopramide in Treating Vomiting in Pediatric Patients With Acute Gastroenteritis: A Network Meta-Analysis

**DOI:** 10.7759/cureus.67902

**Published:** 2024-08-27

**Authors:** Kaushik S Barot, Kalpesh N Vaghasiya, Gaurang H Suhagiya, Aradhana P Singh, Shiza Nadeem, Ahmed Nasir Qureshi, Samina Kutiyana

**Affiliations:** 1 Pediatrics, Shantabaa Medical College and General Hospital Amreli, Amreli, IND; 2 Pediatrics and Child Health, Shantabaa Medical College and General Hospital Amreli, Amreli, IND; 3 Medicine, Jiangsu University, Zhenjiang, CHN; 4 Medicine, Urban Health and Wellness Centre, Surat, IND; 5 Medicine, Islamic International Medical College, Islamabad, PAK; 6 Surgery, Ghurki Trust Teaching Hospital, Lahore, PAK; 7 Cardiology, Mahatma Gandhi Memorial Hospital, Bhojpur, NPL

**Keywords:** vomiting, acute gastroenteritis, metoclopramide, domperidone, ondansetron

## Abstract

This network meta-analysis compared the efficacy of ondansetron, domperidone, and metoclopramide in managing vomiting in pediatric acute gastroenteritis. A comprehensive literature search was conducted across multiple databases, including PubMed, Cochrane Library, Web of Science, and Embase, from their inception to July 25, 2024. Additionally, Google Scholar was searched to identify further relevant studies. In total, 19 randomized controlled trials (RCTs) were included. The primary outcome was cessation of vomiting. The results indicated that ondansetron was significantly more effective than placebo in achieving cessation of vomiting. While domperidone and metoclopramide also showed improved efficacy compared to placebo, these differences were not statistically significant. Ondansetron emerged as the most effective intervention, followed by domperidone and metoclopramide. These findings have significant clinical implications, suggesting that ondansetron should be the preferred antiemetic for pediatric acute gastroenteritis. Its use may reduce the need for intravenous rehydration and hospitalization, potentially improving patient outcomes and reducing healthcare costs. However, the study has limitations, including a lack of data on secondary outcomes and safety profiles of the interventions. Future prospective, multicenter studies are needed to assess both the efficacy and safety of these antiemetics comprehensively in pediatric acute gastroenteritis.

## Introduction and background

Acute gastroenteritis (AGE) in pediatric patients is a common condition characterized by the sudden onset of diarrhea, with or without vomiting, often accompanied by fever, abdominal pain, and dehydration [1]. This condition is predominantly caused by viral infections, though bacteria and parasites can also be responsible [2]. Globally, acute gastroenteritis is a significant cause of morbidity and mortality in children, particularly in developing countries. It accounts for approximately 1.7 billion episodes annually and results in around 525,000 deaths among children under five years of age [3]. The impact of AGE includes severe dehydration, electrolyte imbalances, and, in severe cases, hospitalization or even death if not managed promptly and effectively [4]. The primary treatment approach focuses on rehydration therapy, which can be administered orally or intravenously, depending on the severity of dehydration. Effective management of symptoms like vomiting is crucial to ensure successful rehydration and overall patient comfort [5].

Treatment for AGE often includes antiemetics to control vomiting and facilitate rehydration. Ondansetron, a serotonin 5-HT3 receptor antagonist, is widely used due to its efficacy in reducing vomiting and decreasing the need for intravenous fluids and hospitalization. However, it can rarely contribute to serotonin syndrome, particularly when used in combination with other serotonergic drugs [6]. Domperidone, a dopamine antagonist, enhances gastrointestinal motility and helps manage vomiting but requires careful monitoring due to potential cardiac side effects [7]. Metoclopramide, another dopamine antagonist, also promotes gastric emptying and controls vomiting, though its use is limited by side effects such as extrapyramidal symptoms and sedation [8]. These medications play a crucial role in improving patient outcomes by ensuring effective rehydration and patient comfort.

Understanding the comparative effectiveness and safety of these antiemetic medications is essential for optimizing pediatric care in acute gastroenteritis. A meta-analysis of ondansetron, domperidone, and metoclopramide will provide a comprehensive evaluation of existing clinical data, helping to identify the most effective treatment option. This study aims to systematically review and analyze clinical trials to offer clear, evidence-based recommendations for managing vomiting in pediatric acute gastroenteritis. Ultimately, this will guide healthcare professionals in making informed therapeutic decisions and improving patient outcomes.

## Review

Methodology 

This study was reported following the Preferred Reporting Items for Systematic Reviews and Meta-Analyses (PRISMA) guidelines. This methodological approach ensured a rigorous and systematic evaluation of the available evidence, providing reliable and comprehensive insights into the comparative efficacy and safety of ondansetron, domperidone, and metoclopramide for managing vomiting in pediatric patients with acute gastroenteritis.

Literature Search 

A comprehensive literature search was conducted to identify studies evaluating the efficacy and safety of ondansetron, domperidone, and metoclopramide in treating vomiting in pediatric patients with acute gastroenteritis. The search was performed across electronic databases including PubMed, Cochrane Library, and Embase. Additionally, Google Scholar was used to find further relevant studies. The search strategy utilized a combination of keywords and medical subject headings (MeSH) such as “acute gastroenteritis,” “pediatric,” “children,” “vomiting,” “ondansetron,” “domperidone,” “metoclopramide,” and “antiemetics.” The search covered the period from the inception of the databases to 25th July 2024. Two authors independently conducted the search, with any disagreements resolved through discussion. Reference lists of all included studies were manually screened to identify additional relevant studies.

Study Selection

Studies were selected based on predefined inclusion and exclusion criteria. Inclusion criteria comprised randomized controlled trials (RCTs) evaluating ondansetron, domperidone, or metoclopramide in children with acute gastroenteritis. Included studies had to report the primary outcome of cessation of vomiting. Exclusion criteria included studies not involving pediatric patients, studies without a control group, and those not published in English. Studies focusing on conditions other than acute gastroenteritis, as well as observational studies, reviews, case reports, and case series, were also excluded. Two reviewers independently screened titles and abstracts for relevance and then reviewed full texts to confirm eligibility. Discrepancies were resolved through discussion or consultation with a third reviewer.

Data Extraction

Data were extracted from the selected studies using a standardized form. Extracted information included study characteristics (author, year, country, study design, groups, sample size) and outcomes. Where necessary, authors were contacted to obtain missing data or clarify unclear information. The extracted data were reviewed and cross-checked by the two reviewers to ensure accuracy and consistency.

Data Analysis

In this network meta-analysis, both direct and network meta-analyses were performed. Calculations were carried out using RStudio Version 4.2.2 (Boston, Massachusetts), with treatment effects expressed as odds ratios (ORs) for dichotomous outcomes, accompanied by 95% credible intervals (CIs). Heterogeneity in pairwise meta-analyses was assessed using the I² statistic, which measures the percentage of variability attributable to differences between studies rather than sampling error. Substantial heterogeneity was considered present if the P-value was <0.1 and I² exceeded 50%. Due to inevitable variations between studies, a random-effects model was used in the direct meta-analysis to estimate outcomes. A geometry plot was employed to visually depict all direct comparisons per outcome, with each node representing an intervention. The node-split method was used to assess inconsistency between direct and indirect evidence in closed loops, with a P-value of <0.05 indicating inconsistency. If direct and indirect effects were inconsistent, results were estimated using the inconsistency model; otherwise, the consistency model was applied. Additionally, the surface under the cumulative ranking curve was calculated to rank all interventions. 

Results 

**Figure 1:**
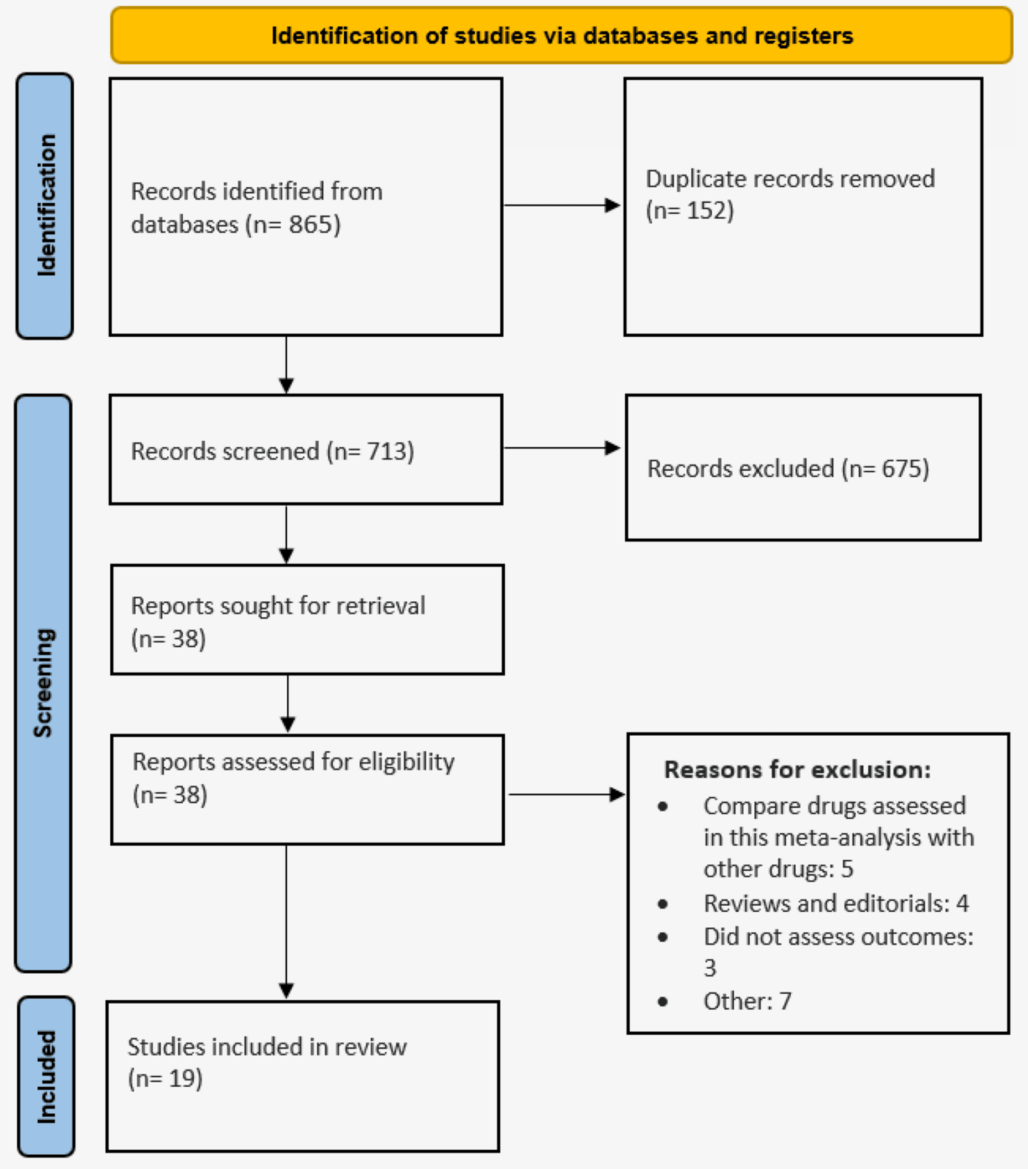
PRISMA flowchart of study selection

We initially identified 865 records in the target databases, of which 152 were duplicates. After screening titles and abstracts, 38 records were retained for full-text review. Ultimately, 19 studies were included in the meta-analysis. Figure 1 presents a PRISMA flowchart depicting the study selection process, and Table 1 details the characteristics of the included studies.

Study Characteristics

Of the 19 included studies, 18 evaluated ondansetron, 12 assessed domperidone, 3 investigated metoclopramide, and 10 included a placebo group. The characteristics of the included studies are detailed in Table 2. All studies were published between 1997 and 2023, with the majority conducted in Pakistan (n=5).

**Table 1:** Characteristics of included studies RCT: randomized control trial.

Author	Year	Design	Region	Groups	Sample Size
Ahmad et al. [9]	2022	RCT	Pakistan	Ondansetron	150
				Domperidone	150
Ahmad et al. [10]	2023	RCT	Pakistan	Ondansetron	40
				Metoclopramide	40
				Domperidone	40
Al-Ansari et al. [11]	2011	RCT	Qatar	Ondansetron	84
				Metoclopramide	83
Bonvanie et al. [12]	2021	RCT	The Netherlands	Ondansetron	87
				Placebo	88
Cubeddu et al. [13]	1997	RCT	Venezuela	Ondansetron	12
				Metoclopramide	12
				Placebo	12
Freedman et al. [14]	2006	RCT	The United States	Ondansetron	107
				Placebo	107
Golshekan et al. [15]	2013	RCT	Iran	Ondansetron	88
				Placebo	88
Hanif et al. [16]	2019	RCT	Pakistan	Ondansetron	123
				Domperidone	117
Ibrahim et al. [17]	2022	RCT	Pakistan	Ondansetron	75
				Domperidone	75
Iqbal et al. [18]	2022	RCT	Pakistan	Ondansetron	123
				Domperidone	117
Kamal et al. [19]	2015	RCT	India	Ondansetron	42
				Domperidone	42
Kita et al. [20]	2015	RCT	Japan	Domperidone	22
				Placebo	29
Leitz et al. [21]	2019	RCT	England	Domperidone	147
				Placebo	145
Marchetti et al. [22]	2016	RCT	Italy	Ondansetron	118
				Domperidone	119
				Placebo	117
Ramsook et al. [23]	2002	RCT	Trinidad	Ondansetron	74
				Placebo	71
Rang et al. [24]	2019	RCT	India	Ondansetron	30
				Placebo	31
Reeves et al. [25]	2002	RCT	The United States	Ondansetron	54
				Placebo	53
Rerksuppaphol [26]	2010	RCT	Thailand	Ondansetron	37
				Placebo	37
Rerksuppaphol [27]	2013	RCT	Thailand	Ondansetron	38
				Domperidone	38
Shah et al. [28]	2013	RCT	Kenya	Ondansetron	60
				Placebo	59

Inconsistency Test 

**Figure 2:**
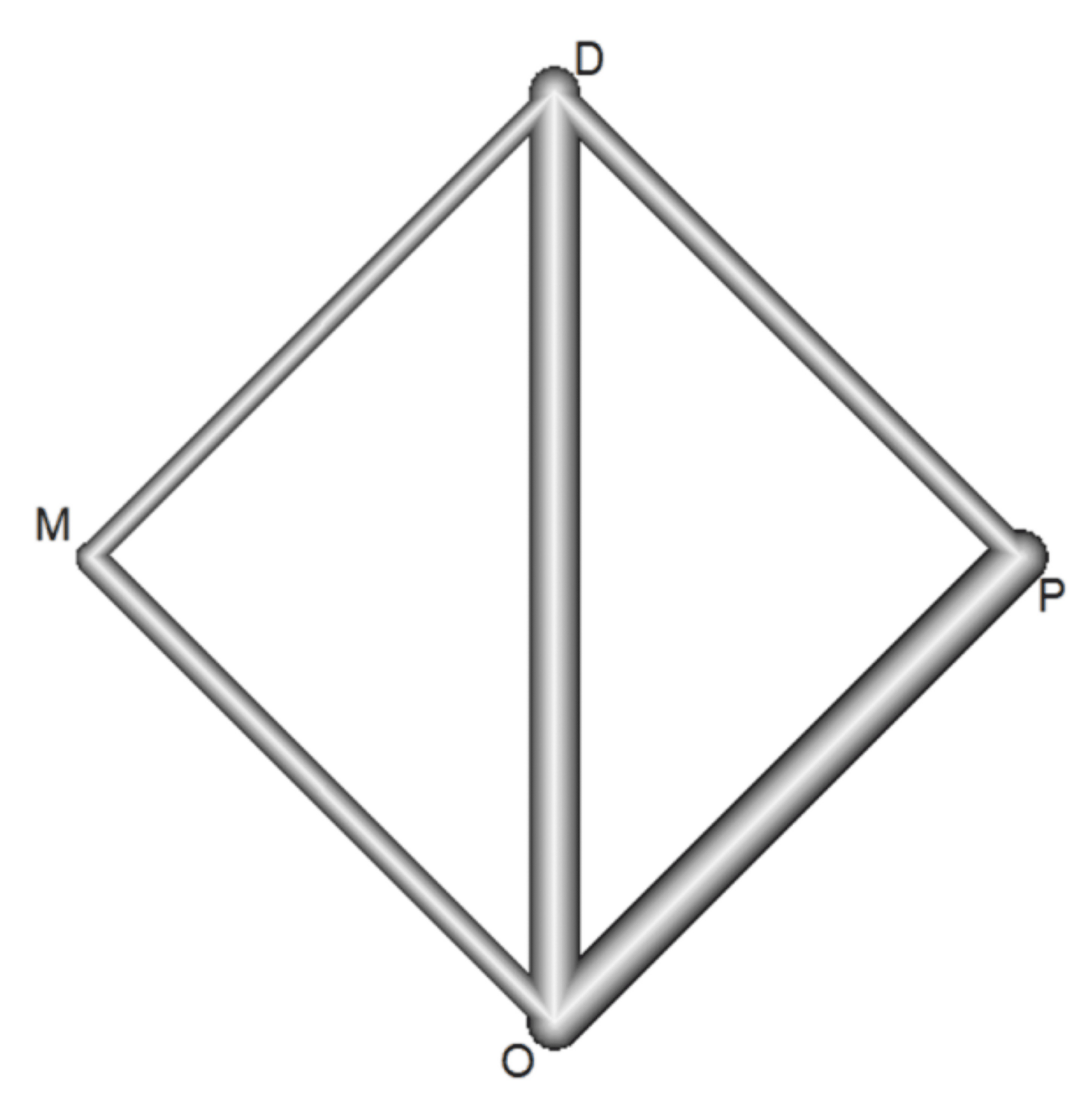
Network graph O: ondansetron, D: domperidone, M: metoclopramide, P: placebo.

A closed loop was identified for cessation of vomiting, as depicted in the network graph (Figure 2). Direct and indirect effects were deemed consistent based on the global consistency test, with a P-value of 0.27 for cessation of vomiting. Local consistency tests were also performed, and all treatment comparisons yielded statistically insignificant P-values (p > 0.05), indicating no inconsistency. Therefore, the consistency model is supported.

Meta-Analysis of Cessation of Vomiting 

**Figure 3:**
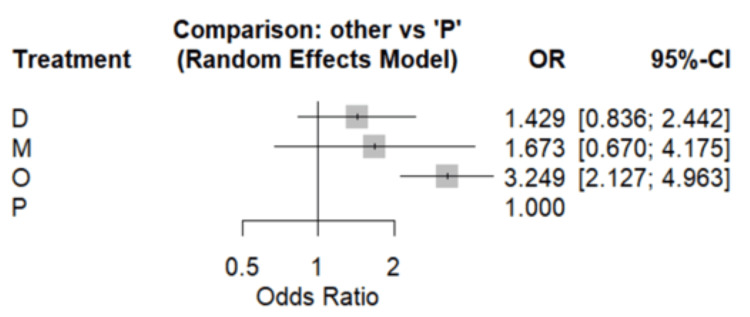
Forest plot comparing all drugs with placebo O: ondansetron, D: domperidone, M: metoclopramide, P: placebo, OR: odds ratio, CI: confidence interval.

We compared the cessation of vomiting among four groups: ondansetron, domperidone, metoclopramide, and placebo. Compared to the placebo group, cessation of vomiting was significantly higher in patients receiving ondansetron. Additionally, cessation was higher in domperidone and metoclopramide compared to patients receiving placebo but the difference was insignificant as shown in Figure 3. Table 2 presents the results of the comparison of all treatments with each other. Surface under the cumulative ranking curve (SUCRA) score showed that among all the treatments, ondansetron was the best in terms of cessation of vomiting (SUCRA Score: 0.92) followed by domperidone (SUCRA score: 0.58) and metoclopramide (SUCRA score: 0.48).

**Table 2:** Comparison of cessation of vomiting among different treatment groups All values are presented as odds ratio (95% confidence interval).

Groups	Groups			
	Domperidone	Metoclopramide	Ondansetron	Placebo
Domperidone	1	0.85 (0.35-2.05)	0.44 (0.28-0.69)	1.43 (0.84-2.44)
Metoclopramide	1.17 (0.49-2.82)	1	0.51 (0.22-1.18)	1.67 (0.67-4.17)
Ondansetron	2.27 (1.44-3.59)	1.94 (0.85-4.45)	1	3.25 (2.13-4.96)
Placebo	0.70 (0.41-1.20)	0.60 (0.24-1.49)	0.31 (0.20-0.47)	1

Discussion

In this network meta-analysis, we compared the efficacy of ondansetron, domperidone, and metoclopramide in terms of cessation of vomiting in patients with acute gastroenteritis. The present meta-analysis reported that ondansetron is the best intervention for cessation of vomiting. A meta-analysis conducted by Laura et al. also reported that ondansetron is the only intervention that revealed an effect on the cessation of vomiting [29]. Another meta-analysis conducted in 2022 found that, compared to domperidone, ondansetron was more effective in achieving cessation of vomiting in children with gastroenteritis [30]. This is possibly because when ondansetron is taken orally, it is absorbed in the gastrointestinal tract and acts as a "serotonin 5-HT3 receptor antagonist," suppressing the brain's vomiting centers and blocking afferent depolarization of peripheral vagal nerves in the intestine, which may cause emesis responses in gastroenteritis patients [31, 32]. As ondansetron is thought to lessen emesis, it may increase the oral intake of fluids, thereby reducing the need for hospitalization and intravenous rehydration [33].

Dopamine antagonists such as metoclopramide and domperidone are less effective and have a higher chance of side effects. Domperidone’s main effect is to improve motility in the gastrointestinal tract; nevertheless, it has a less targeted antiemetic effect and may cause cardiac arrhythmias, especially in younger patients [18]. Metoclopramide similarly increases gastrointestinal motility, although its usage in pediatric patients is limited due to significant adverse effects, such as extrapyramidal symptoms, including involuntary movements [32].

The use of antiemetics in children with acute gastroenteritis has generated debate. The use of antiemetics is not advised by groups like the American Academy of Pediatrics [34], the National Institute for Health and Care Excellence, or the World Health Organization [35]. Ondansetron, on the other hand, is recommended by the European Society for Pediatric Gastroenterology, Hepatology, and Nutrition clinical practice guidelines (CPGs) [36]. This discrepancy may result from the previous organizations' outdated CPGs, which neglected to consider new information. Our findings could therefore be very important for upcoming CPG updates.

This research highlights ondansetron's superior efficacy and safety compared to domperidone and metoclopramide in managing vomiting in children with acute gastroenteritis. Clinically, these findings suggest that ondansetron should be the preferred antiemetic, potentially leading to updates in treatment guidelines. By effectively reducing vomiting, ondansetron may decrease the need for intravenous rehydration and hospital admissions, improving patient outcomes and reducing healthcare costs. The research also underscores the importance of prioritizing safer medications in pediatric care, prompting clinicians to reconsider the use of domperidone and metoclopramide due to their associated risks and lower efficacy.

There are limitations to the current meta-analysis. First, every study examined how medications affect vomiting cessation; however, most studies did not evaluate additional outcomes, such as the need for intravenous fluids and subsequent rehospitalization. Additionally, the lack of experimental investigations comparing the safety profiles of domperidone and ondansetron meant that the current meta-analysis was unable to evaluate adverse events across the two groups. To obtain data for use in the clinical context, prospective, multicenter studies in the future are required to assess the safety outcomes of domperidone compared to ondansetron.

## Conclusions

This meta-analysis offers robust evidence supporting ondansetron's superiority in treating vomiting among pediatric patients with acute gastroenteritis. The data revealed that ondansetron significantly outperformed domperidone, metoclopramide, and placebo in effectively ceasing vomiting, making it the most effective antiemetic option for this condition. These results carry substantial clinical implications, advocating for ondansetron to be the first-line treatment in managing pediatric gastroenteritis-related vomiting. Moreover, the study highlights the critical need for revising and updating clinical practice guidelines to incorporate the most recent evidence, ensuring that pediatric care remains grounded in the best available research. By prioritizing evidence-based practices, healthcare providers can enhance patient outcomes, reduce the need for more invasive treatments, and optimize resource utilization in the management of pediatric gastroenteritis. This underscores the broader importance of continually revisiting and refining treatment protocols in light of new, high-quality evidence.

## References

[1] Liao Y, Hong X, Wu A (2021). Global prevalence of norovirus in cases of acute gastroenteritis from 1997 to 2021: an updated systematic review and meta-analysis. Microb Pathog.

[2] Guarino A, Aguilar J, Berkley J (2020). Acute gastroenteritis in children of the world: what needs to be done?. J Pediatr Gastroenterol Nutr.

[3] Omatola CA, Ogunsakin RE, Onoja AB (2024). Enteropathogenic viruses associated with acute gastroenteritis among African children under 5 years of age: a systematic review and meta-analysis. J Infect.

[4] Szajewska H, Guarino A, Hojsak I (2020). Use of probiotics for the management of acute gastroenteritis in children: an update. J Pediatr Gastroenterol Nutr.

[5] Burke RM, Mattison CP, Marsh Z (2021). Norovirus and other viral causes of medically attended acute gastroenteritis across the age spectrum: results from the medically attended acute gastroenteritis study in the United States. Clin Infect Dis.

[6] Fonseca NM, Pedrosa LR, Melo N, Oliveira RÁ (2020). Effect of palonosetron, ondansetron and dexamethasone in the prevention of postoperative nausea and vomiting in video cholecystectomy with total venous anesthesia with propofol-remifentanil - randomized clinical trial (Article in Portuguese). Braz J Anesthesiol.

[7] Heckroth M, Luckett RT, Moser C, Parajuli D, Abell TL (2021). Nausea and vomiting in 2021: a comprehensive update. J Clin Gastroenterol.

[8] Orhan A, Nguyen C, Chan A, Herrstedt J (2024). Pharmacokinetics, pharmacodynamics, safety, and tolerability of dopamine-receptor antagonists for the prevention of chemotherapy-induced nausea and vomiting. Expert Opin Drug Metab Toxicol.

[9] Ahmad T, Zarafshan U, Sahar B (2022). Comparison of ondansetron versus domperidone for treating vomiting in acute gastroenteritis in children at a resource limited setting of South Punjab, Pakistan. Pak J Med Sci.

[10] Ahmad M, Rawat A, Farrukh S, Haq I, Kumar Mandal A, Syed A, Sajid M (2023). Comparative analysis of oral ondansetron, metoclopramide, and domperidone for managing vomiting in children with acute gastroenteritis. Cureus.

[11] Al-Ansari K, Alomary S, Abdulateef H, Alshawagfa M, Kamal K (2011). Metoclopramide versus ondansetron for the treatment of vomiting in children with acute gastroenteritis. J Pediatr Gastroenterol Nutr.

[12] Bonvanie IJ, Weghorst AA, Holtman GA (2021). Oral ondansetron for paediatric gastroenteritis in primary care: a randomised controlled trial. Br J Gen Pract.

[13] Cubeddu LX, Trujillo LM, Talmaciu I (1997). Antiemetic activity of ondansetron in acute gastroenteritis. Aliment Pharmacol Ther.

[14] Freedman SB, Adler M, Seshadri R, Powell EC (2006). Oral ondansetron for gastroenteritis in a pediatric emergency department. N Engl J Med.

[15] Golshekan K, Badeli H, Rezaieian S, Mohammadpour H, Hassanzadehrad A (2013). Effect of oral ondansetron on decreasing the vomiting associated with acute gastroenteritis in Iranian children. Iran J Pediatr.

[16] Hanif H, Jaffry H, Jamshed F, Amreek F, Kumar N, Hussain W, Rizwan A (2019). Oral ondansetron versus domperidone for acute gastroenteritis associated vomiting in young children. Cureus.

[17] Ibrahim FA, Farhat AN, Butt NA, Sadiq MA, Rafiq AB, MD C (2022). Ondansetron vs domperidone in the treatment of vomiting in children with acute diarrhea: a randomized, double-blind study. Pakistan J Med Health Sci.

[18] Iqbal MB, Anjum BA, Haider N, Nawab I, Masood A, Krishin J (2022). Comparison of efficacy of ondansteron versus domperidone for management of vomiting in children with acute gastroenteritis. Rawal Med J.

[19] Kamal SS, BLK P, Pathapati RM, Buchineni M (2015). Clinical outcome with single dose ondansetron versus domperidone in paediatric gastroenteritis-our experience. J Med Sci Clin Res.

[20] Kita F, Hinotsu S, Yorifuji T (2015). Domperidone with ORT in the treatment of pediatric acute gastroenteritis in Japan: a multicenter, randomized controlled trial. Asia Pac J Public Health.

[21] Leitz G, Hu P, Appiani C, Li Q, Mitha E, Garces-Sanchez M, Gupta R (2019). Safety and efficacy of low-dose domperidone for treating nausea and vomiting due to acute gastroenteritis in children. J Pediatr Gastroenterol Nutr.

[22] Marchetti F, Bonati M, Maestro A (2016). Oral ondansetron versus domperidone for acute gastroenteritis in pediatric emergency departments: multicenter double blind randomized controlled trial. PLoS One.

[23] Ramsook C, Sahagun-Carreon I, Kozinetz CA, Moro-Sutherland D (2002). A randomized clinical trial comparing oral ondansetron with placebo in children with vomiting from acute gastroenteritis. Ann Emerg Med.

[24] Rang NN, Chanh TQ, My PT, Tien TT (2019). Single-dose intravenous ondansetron in children with gastroenteritis: a randomized controlled trial. Indian Pediatr.

[25] Reeves JJ, Shannon MW, Fleisher GR (2002). Ondansetron decreases vomiting associated with acute gastroenteritis: a randomized, controlled trial. Pediatrics.

[26] Rerksuppaphol S, Rerksuppaphol L (2010). Efficacy of intravenous ondansetron to prevent vomiting episodes in acute gastroenteritis: a randomized, double blind, and controlled trial. Pediatr Rep.

[27] Rerksuppaphol S, Rerksuppaphol L (2013). Randomized study of ondansetron versus domperidone in the treatment of children with acute gastroenteritis. J Clin Med Res.

[28] Shah AA (2015). Effect of Oral Ondansetron in Children Presenting with Acute Diarrhoeal Illness and Vomiting With Some Dehydration (Thesis). Thesis.

[29] Niño-Serna LF, Acosta-Reyes J, Veroniki AA, Florez ID (2020). Antiemetics in children with acute gastroenteritis: a meta-analysis. Pediatrics.

[30] Aisha F, Bhagwani K, Ijaz H (2022). Comparison of the effectiveness of ondansetron and domperidone in cessation of vomiting in children presenting with acute gastroenteritis: a meta-analysis. Cureus.

[31] Xu M, Rieder M (2014). A supplementary home dose of oral ondansetron given in anticipation of recurrent emesis in paediatric acute gastroenteritis. Paediatr Child Health.

[32] Tomasik E, Ziółkowska E, Kołodziej M, Szajewska H (2016). Systematic review with meta-analysis: ondansetron for vomiting in children with acute gastroenteritis. Aliment Pharmacol Ther.

[33] Gray JM, Maewal JD, Lunos SA, Furnival RA, Hendrickson MA (2020). Ondansetron prescription for home use in a pediatric emergency department. Pediatr Emerg Care.

[34] (2005). The treatment of diarrhoea. A manual for physicians and other senior health workers, 4th Revision. World Health Organization.

[35] National Collaborating Centre for Women's and Children's Health (UK) (2009). Diarrhoea and Vomiting Caused by Gastroenteritis. Diagnosis, Assessment and Management in Children Younger than 5 Years. Diarrhoea and Vomiting Caused by Gastroenteritis. Diagnosis, Assessment and Management in Children Younger Than 5 Years.

[36] Guarino A, Ashkenazi S, Gendrel D, Lo Vecchio A, Shamir R, Szajewska H (2014). European Society for Pediatric Gastroenterology, Hepatology, and Nutrition/European Society for Pediatric Infectious Diseases evidence-based guidelines for the management of acute gastroenteritis in children in Europe: update 2014. J Pediatr Gastroenterol Nutr.

